# Prediction of Long-Term Physical, Mental, and Cognitive Problems Following Critical Illness: Development and External Validation of the PROSPECT Prediction Model*

**DOI:** 10.1097/CCM.0000000000006073

**Published:** 2023-12-15

**Authors:** Dries van Sleeuwen, Marieke Zegers, Jordache Ramjith, Juliette K. Cruijsberg, Koen S. Simons, Daniëlle van Bommel, Dominique Burgers-Bonthuis, Julia Koeter, Laurens L. A. Bisschops, Inge Janssen, Thijs C. D. Rettig, Johannes G. van der Hoeven, Floris A. van de Laar, Mark van den Boogaard

**Affiliations:** 1 Department of Primary and Community Care, Radboud University Medical Center, Nijmegen, The Netherlands.; 2 Department of Intensive Care, Radboud University Medical Center, Nijmegen, The Netherlands.; 3 Department for Health Evidence, Biostatistics Research Group, Radboud University Medical Center, Nijmegen, The Netherlands.; 4 IQ Healthcare, Radboud University Medical Center, Nijmegen, The Netherlands.; 5 Department of Intensive Care Medicine, Jeroen Bosch Hospital, ’s Hertogenbosch, The Netherlands.; 6 Department of Intensive Care Medicine, Bernhoven Hospital, Uden, The Netherlands.; 7 Department of Intensive Care Medicine, Rijnstate Hospital, Arnhem, The Netherlands.; 8 Department of Intensive Care Medicine, CWZ, Nijmegen, The Netherlands.; 9 Department of Intensive Care Medicine, Maasziekenhuis, Boxmeer, The Netherlands.; 10 Department of Anesthesiology, Intensive Care Medicine, and Pain Medicine, Amphia Hospital, Breda, The Netherlands.

**Keywords:** critical illness, intensive care, prediction, preventive medicine

## Abstract

**OBJECTIVES:**

ICU survivors often suffer from long-lasting physical, mental, and cognitive health problems after hospital discharge. As several interventions that treat or prevent these problems already start during ICU stay, patients at high risk should be identified early. This study aimed to develop a model for early prediction of post-ICU health problems within 48 hours after ICU admission.

**DESIGN:**

Prospective cohort study in seven Dutch ICUs.

**SETTING/PATIENTS:**

ICU patients older than 16 years and admitted for greater than or equal to 12 hours between July 2016 and March 2020.

**INTERVENTIONS:**

None.

**MEASUREMENTS AND MAIN RESULTS:**

Outcomes were physical problems (fatigue or ≥ 3 new physical symptoms), mental problems (anxiety, depression, or post-traumatic stress disorder), and cognitive impairment. Patient record data and questionnaire data were collected at ICU admission, and after 3 and 12 months, of 2,476 patients. Several models predicting physical, mental, or cognitive problems and a composite score at 3 and 12 months were developed using variables collected within 48 hours after ICU admission. Based on performance and clinical feasibility, a model, PROSPECT, predicting post-ICU health problems at 3 months was chosen, including the predictors of chronic obstructive pulmonary disease, admission type, expected length of ICU stay greater than or equal to 2 days, and preadmission anxiety and fatigue. Internal validation using bootstrapping on data of the largest hospital (*n* = 1,244) yielded a *C*-statistic of 0.73 (95% CI, 0.70–0.76). External validation was performed on data (*n* = 864) from the other six hospitals with a *C*-statistic of 0.77 (95% CI, 0.73–0.80).

**CONCLUSIONS:**

The developed and externally validated PROSPECT model can be used within 48 hours after ICU admission for identifying patients with an increased risk of post-ICU problems 3 months after ICU admission. Timely preventive interventions starting during ICU admission and follow-up care can prevent or mitigate post-ICU problems in these high-risk patients.

**KEY POINTS**

**Question**: Can we predict post-ICU health problems within 48 hours after ICU admission for early prevention or treatment?

**Findings**: The developed and externally validated PROSPECT model with five predictors reliably predicts patients’ risk for health problems at 3 months after ICU admission. The predictors are as follows: chronic obstructive pulmonary disease, admission type, expected length of ICU stay of greater than or equal to 2 days, and preadmission anxiety and fatigue.

**Meaning**: As several interventions for preventing or mitigating post-ICU health problems already start in the ICU, early identification of high-risk patients for post-ICU problems is necessary. The PROSPECT model identifies high-risk patients within 48 hours after ICU admission.

As more patients survive ICU treatment thanks to advances in critical care medicine (1), the number of ICU survivors experiencing long-lasting health problems is increasing. These long-lasting multiple organ sequelae are described as chronic critical illness and have impact on work, daily functioning, and quality of life (QoL) (1–6).

To prevent or mitigate these problems, attention to preventive interventions and recovery programs is growing (7–11). Early identification of high-risk patients is crucial as several known interventions already start during ICU stay (12–15). However, prediction models for early selection of ICU survivors with a high risk for health problems post-ICU lack, and therefore, invitations for follow-up care are now largely based on expert opinion (16, 17). A prediction model not only practically selects patients for post-ICU care but also provides clinicians more insight into which patients develop post-ICU health problems. This knowledge can be used to further fuel discussions of patients’ post-ICU prognosis with patients, caregivers, and healthcare providers at ICU admission. Therefore, developing a prediction model for post-ICU problems is highly prioritized on research agendas (18–21).

The Society of Critical Care Medicine already recommended the development of a prediction model for post-ICU health problems, including physical, mental, and cognitive functioning, and to take pre-ICU functioning into account (19). A systematic review only found three existing prediction models to predict long-term impairments after critical illness. However, these models were considered to be at high risk of bias, did not include all three health domains (physical, mental, and cognitive functioning), and have not been externally validated (18). Assessment of post-ICU morbidity risk is recommended 2–3 months after ICU discharge; however, problems may also emerge later (6). Furthermore, a prediction model needs to be easy to use in clinical practice (22).

Accordingly, the aim of this study was to develop and externally validate a prediction model for post-ICU physical, mental, or cognitive health problems that can be easily used in clinical practice shortly after ICU admission.

## MATERIALS AND METHODS

### Study Design

Data for this study were obtained from an ongoing multicenter prospective cohort study (MONITOR-IC study), in which long-term outcomes of ICU patients are followed up after ICU admission (ClinicalTrials.gov: NCT03246334). The MONITOR-IC study was approved by the local ethics committee of the Radboud University Medical Center, Committee on Research Involving Human Subjects, region Arnhem-Nijmegen, The Netherlands (2016-2724) and conducted in accordance with the declaration of Helsinki (23). For this study, the Transparent Reporting of a multivariable prediction model for Individual Prognosis Or Diagnosis guidelines were applied (**Appendix 1**, http://links.lww.com/CCM/H432) (24).

### Study Population

In the MONITOR-IC study, data of ICU patients 16 years old or older and admitted for at least 12 hours to one of the seven participating hospitals in the Netherlands were collected. ICU patients (medical, elective surgical, and emergency surgical) admitted between July 2016 and March 2020 (pre-COVID-19) were included in the present study. The largest hospital offered post-ICU care by means of an outpatient clinic visit after hospital discharge at the patient’s own request. Post-ICU care in the other hospitals varied ranging from no post-ICU care or only a telephone call to an outpatient clinic visit after hospital discharge. Patients were excluded when they had a life expectancy of less than 48 hours or could not read or speak the Dutch language.

### Outcomes

Physical domain was defined as extreme fatigue defined by a score of greater than 37 on the Checklist Individual Strength—fatigue subscale (CIS-8) (25, 26) or three or more physical problems objectified by a list of 30 symptoms and were present if at least one symptom was moderate or severe.

Mental domain was defined as symptoms of post-traumatic stress disorder defined by a mean of all questions greater than or equal to 1.75 on the Impact of Event Scale (IES)-6 (27, 28), or anxiety and depression symptoms defined by a score of greater than or equal to 8 on the Hospital Anxiety and Depression Scale (HADS) subscales (29, 30).

Cognitive domain was defined as cognitive impairment with a score of greater than or equal to 43 on the abbreviated Cognitive Failure Questionnaire-14 (31).

Finally, a general outcome was created as a composite score of physical, mental, and cognitive domains using the same criteria as the individual outcome measures. So, patients were categorized as positive for post-ICU health problems, in general, if they had one or more positive scores in the physical, mental, and/or cognitive domain.

### Data Collection

Patients, or their relatives in case patients were not able to fill in the questionnaire themselves, completed a baseline questionnaire concerning patients’ health status before ICU admission and 3 and 12 months after ICU admission. Elective surgical patients received the baseline questionnaire at the preoperative outpatient clinic and completed the questionnaire a few days before their ICU admission. For medical and emergency surgical patients, this was not possible and they, therefore, received the baseline questionnaire while in the ICU. These patients, or their proxies, were then asked to rate patients’ health status in retrospect, recalling their health status before ICU admission. Depending on their preferences, patients received the questionnaires online or on paper. For the baseline measurement, a reminder was sent after 4 weeks and a reminder by telephone was provided 2 weeks later if necessary. For the 3- and 12-month questionnaires, reminders were sent after 2 and 4 weeks. Patient record data were collected in the first 24 hours of the ICU admission (32).

### Candidate Predictors

In total, 18 candidate predictors were selected based on the results of previous MONITOR-IC research (3). All definitions of candidate predictors can be found in **Appendix 2** (http://links.lww.com/CCM/H432). Linear effects of a scaled Acute Physiology and Chronic Health Evaluation (APACHE) IV score on post-ICU health problems were also fitted in a logistic regression model. Nonlinear effects of this scaled APACHE IV score with penalized splines were modeled using a generalized additive model from the binomial family with a logit link (33).

### Statistical Analysis

Multiple prediction models were developed: three separate models to predict physical, mental, and cognitive problems, respectively, and one model to predict post-ICU health problems, in general, as a composite score: a combined score for physical, mental, or cognitive problems. These outcomes were predicted at either 3 or 12 months after ICU admission. In addition, models with time as a variable were also developed for each outcome, resulting in a total of 12 different models (**Appendix 3**, http://links.lww.com/CCM/H432). To develop the models, data of the largest hospital were used, comprising two-thirds of the included patients. In case of missing values in the CIS-8, HADS, and 36-Item Short Form Health Survey (SF-36) scales, these were imputed using the half rule (34). Missing values in the IES-Revised were replaced with the individual mean, provided that 75% of the items were completed. For other variables, only full cases were used, and missing cases were omitted depending on the timeframe and outcome of each model (**Appendix 4**, http://links.lww.com/CCM/H432). For these variables, complete case analysis was used because most of them are registered by default at ICU admission as part of the Dutch National Institute for Health and Care Excellence (NICE) registry, so there were very few missing values to expect in advance. As the outcome measures were dichotomous, multivariable logistic regression analysis was used to develop the models. To internally validate the models, a total of 1,000 bootstrap samples were taken from the original data. To further reduce the number of suitable candidate predictors, best subsets regression analysis was used to evaluate smaller subsets of models after a backward elimination selection procedure. All developed models were discussed during multiple meetings by a panel of three ICU clinicians (K.S., J.K., L.L.A.B.), an ICU nurse (M.v.B.), a family physician (F.A.v.L.), and a health scientist (M.Z.) to reach consensus on the best discriminating model suitable for clinical practice. The selection criteria for the “best model” were the balance between the total number of input variables, the feasibility of obtaining the variables in clinical practice, and the performance of the models expressed in concordance (*C*-statistic). The *C*-statistic varies between 0.5 and 1.0 for sensible models (the higher, the better). The predictive ability of the model can be considered distinctive with a *C*-statistic around 0.80 (35–38).

Subsequently, the chosen model was externally validated with data from the remaining six hospitals. Development and validation cohorts were created based on hospitals rather than randomization on patient level to obtain a better generalizability after external validation. Calibration was assessed graphically by plotting the observed outcome frequencies against the mean predicted outcome probabilities or risks, within subgroups of patients that were ranked by increasing estimated probability (39). All analyses were performed with R software, Version 3.6.2 (R Foundation for Statistical Computing, Vienna, Austria) (packages haven, dplyr, DescTools, Hmisc, boot, bestglm, xlsx, rms, predtools, magrittr, cutpointr, and ggplot2).

## RESULTS

### Study Population

**Figure 1.**
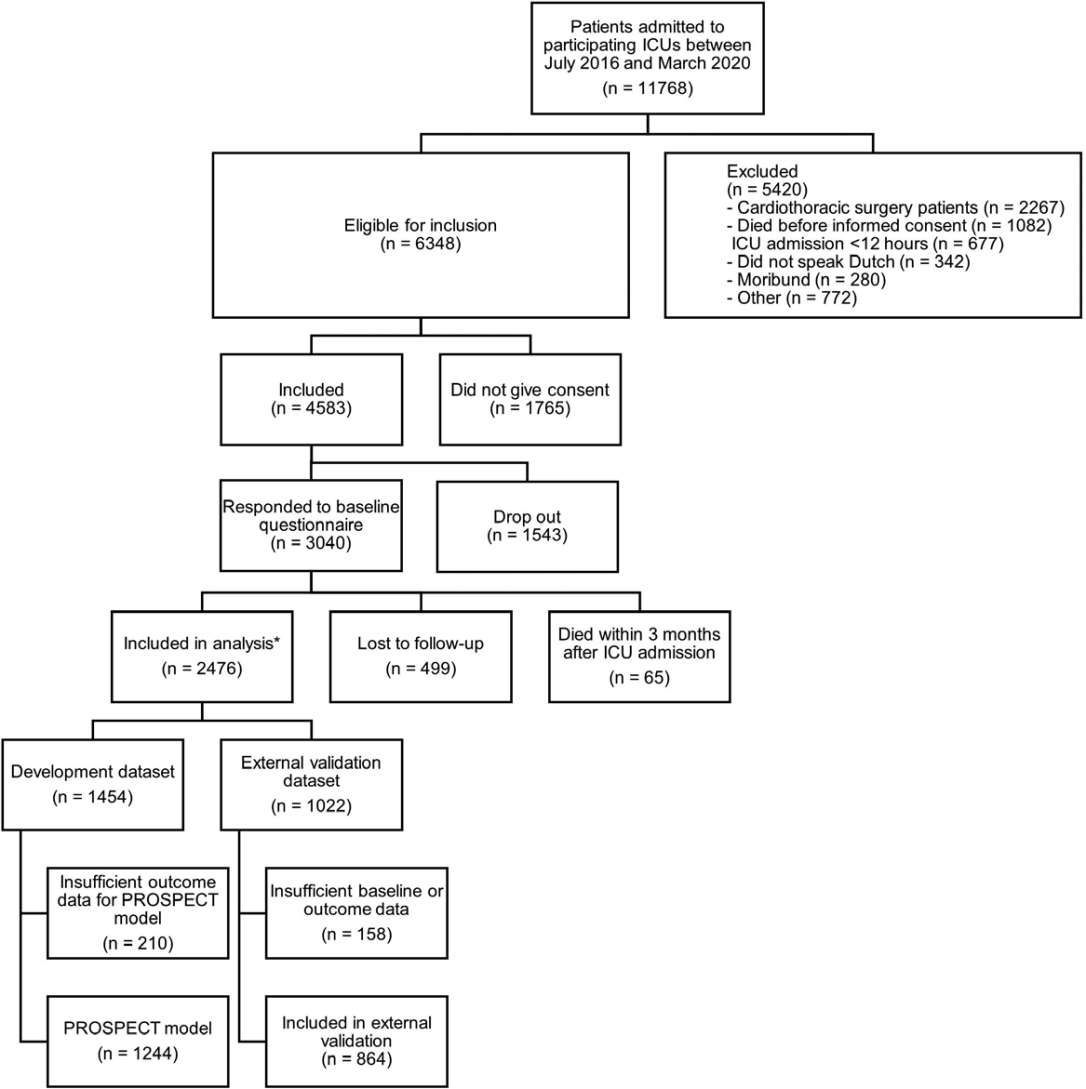
Flowchart of participants. All models were developed from the development dataset (*n* = 1,454). Because this dataset contained some missing values for specific variables for specific time outcomes (Appendix 4, http://links.lww.com/CCM/H432), the total number of patients enrolled in the development of each particular model slightly differed. For the post-ICU three months prediction (PROSPECT) model, 1,244 patients were enrolled in the development. *Responded to 3- and/or 12-mo follow-up questionnaire.

In total, 11,768 patients were admitted to the participating ICUs, and of those, 6,348 patients (54%) were eligible for inclusion (**Fig. 1**). If patients did not complete the 3 or 12 months questionnaire, or died in the follow-up period, their data were not used for the 3 and 12 months models, respectively. In total, 77.8% of the baseline questionnaires were completed by patients, otherwise by proxies. All models were developed from the development dataset (*n* = 1,454) (**Appendix 5**, http://links.lww.com/CCM/H432). The total number of missing values, and as such patients, varied slightly per model because different timeframes and outcome measures were used for different models. Missing candidate predictors are shown in Appendix 4 (http://links.lww.com/CCM/H432). For the post-ICU three months prediction (PROSPECT) model, 1,244 patients were enrolled in the development set. For the external validation, 1,022 patients were included, and of them, 864 patients could be enrolled in the external validation set (Fig. 1). Characteristics of patients included and excluded for the development and validation of the PROSPECT model are shown in **Appendix 6** (http://links.lww.com/CCM/H432).

Three months after ICU admission, the prevalence of physical, mental, or cognitive problems was 60.5%, 38.4%, and 12.0%, respectively. The prevalence of post-ICU health problems, in general, was 69.7%. The prevalence of baseline variables in patients with and without post-ICU health problems is shown in **Appendix 7** (http://links.lww.com/CCM/H432). Twelve months after ICU admission, the prevalence of physical, mental, or cognitive problems was 50.4%, 36.2%, and 10.2%, respectively, and for post-ICU health problems, in general, 59.6%.

### Choice of Model and Best Subset Regression Analysis

All developed models for predicting physical, mental, and cognitive domains for 3 and 12 months, as well as the combined domains, are summarized in Appendix 3 (http://links.lww.com/CCM/H432). There was no evidence of multicollinearity, with variance inflation factors of less than 2.3 for all candidate predictors. Notably, frailty was associated with negative regression coefficients and disease severity (APACHE score) with near zero values. We found that a nonlinear model does not show improvement in the explanation of the association between the APACHE IV score and the log-odds of post-ICU health problems. The best discriminating model was the model predicting mental symptoms 3 months post-ICU (model M3, subtype 7 [**Appendix 3, e-Table S3a-k**, **m**, and **n**, http://links.lww.com/CCM/H432]) and had a C-statistics of 0.79 (95% CI, 0.77–0.81). The expert panel preferred the model predicting health problems, in general, 3 months post-ICU (Model Gen3, subtype 6 [**Appendix 3, e-Table S3l**, http://links.lww.com/CCM/H432]). The expert panel agreed that a 3-month prediction model is most feasible for clinicians for early treatment to prevent post-ICU problems. The model chosen as best fitting for clinical practice (**Table 1**) had five predictors (chronic obstructive pulmonary disease [COPD], admission type, expected length of ICU stay of greater than or equal to 2 days, and preadmission anxiety and fatigue). This model, called the PROSPECT model, had a *C*-statistic of 0.73 (95% CI, 0.70–0.76). A formula of this model, including a calculation example, is shown in **Appendix 8** (http://links.lww.com/CCM/H432).

**TABLE 1.** Regression Coefficients, Model Performance, and Cutoff Points of the PROSPECT Model

Variables		Regression Coefficient		
Intercept		–0.86043		
Length of ICU stay ≥ 2 d				
Yes/no		0.39217		
Chronic obstructive pulmonary disease				
Yes/no		0.621616		
Admission type (ref. medical admission)				
Emergency surgical		0.438867		
Admission type (ref. medical admission)				
Elective surgical		–0.33547		
Anxiety				
Hospital Anxiety and Depression Scale-Anxiety score		0.140676		
Fatigue				
Checklist Individual Strength—fatigue subscale score		0.032472		
Statistics	Internal Validation	External Validation		
Likelihood χ^2^	175.16	171.35		
Nagelkerke *R*^2^	0.19	0.25		
*C*-statistic (95% CI)	0.73 (0.70–0.76)	0.77 (0.73–0.80)		
Brier score	0.18	0.18		
Hosmer-Lemeshow χ^2^ (*p*)	12.68 (0.12)	4.55 (0.81)		
Cutoff Points (%)	Sensitivity (%)	Specificity (%)	Positive LR	Negative LR
40.0	0.77	0.58	1.85	0.39
50.0	0.74	0.60	1.87	0.43
60.0	0.71	0.65	2.02	0.44
70.0	0.68	0.68	2.14	0.47
80.0	0.64	0.71	2.23	0.50
90.0	0.61	0.74	2.37	0.52
100.0	0.58	0.78	2.62	0.54

LR = likelihood ratio, ref = reference category.

### Internal and External Validation

**Figure 2.**
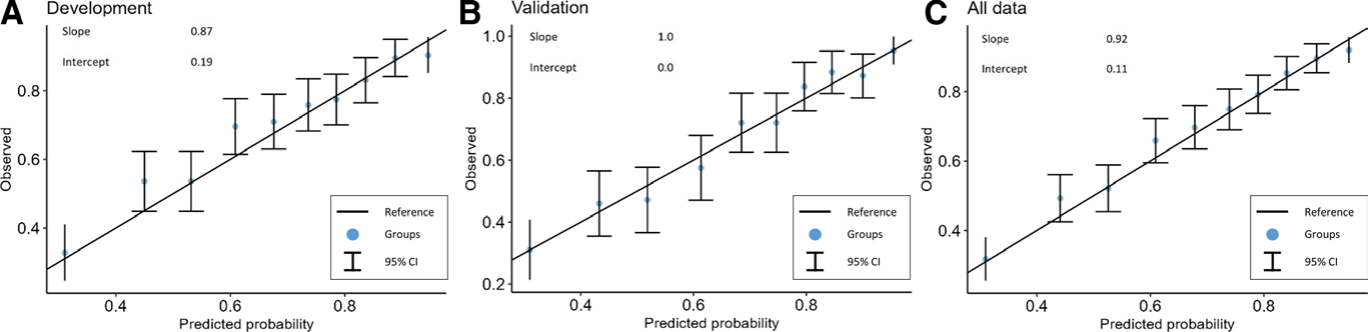
Calibration. Calibration plots for development dataset (**A**), validation dataset (**B**), and all data (**C**).

Internal validation with 1,000 bootstrap samples (optimism *R*^2^: 0.0005; slope: –0.0044) (**Appendix 9**, http://links.lww.com/CCM/H432) of the best-fit PROSPECT model showed adequate fit determined by the model’s intercept and slope (**Fig. 2**). External validation of the best-fit model in the remaining six hospitals (*n* = 864) showed a *C*-statistic of 0.77 (95% CI, 0.73–0.80). The model’s performance is shown in Table 1, and calibration graphs are shown in Figure 2. The calibration graph of the internal validation and external validation dataset combined showed a slope of 0.92 and an intercept of 0.11. Given this slope and intercept, recalibration was considered not to be required. Performance at different risk scores for predicting post-ICU health problems is shown in Table 1. A sensitivity and specificity plot is shown in **Appendix 10** (http://links.lww.com/CCM/H432) and visualization of the spread of the predicted probabilities is in **Appendix 11** (http://links.lww.com/CCM/H432). A subgroup analysis was performed for developing the prediction model without elective surgical patients, but the variable selection and model performance were quite similar to the PROSPECT model (**Appendix 12**, http://links.lww.com/CCM/H432).

## DISCUSSION

Within 48 hours after ICU admission, the PROSPECT model predicts post-ICU health problems 3 months after ICU admission using five predictors (COPD, admission type, expected length of ICU stay of ≥ 2 d, and preadmission anxiety and fatigue). Internal and external validation yielded a *C*-statistic of 0.73 and 0.77, which can be considered as distinctive (35). To our knowledge, this is the first externally validated prediction model predicting post-ICU health problems and the first model using physical, mental, and cognitive functioning as outcome measure.

The discriminative power of the PROSPECT model was comparable to a previously developed model that predicted mental health problems only, and additionally, this model was not externally validated (40). Also, two other models, which predicted only physical functioning, showed a slightly better discrimination, but were developed with a much smaller sample size and were not externally validated (41, 42). The predictors in the best subset prediction model, in this study, are in line with previous long-term outcome research findings, in which pre-ICU mental health problems were strongly associated with post-ICU health problems (43). A noteworthy result was the negative regression coefficient of frailty and the near zero values for the regression coefficients for disease severity (APACHE score). A possible explanation could be that a higher frailty baseline score is associated with being becoming less frail, as previous research showed (44), and the severity of illness score with less post-ICU problems. These findings, however, could also be by cause of the exclusion of terminally ill patients in our study and the complete case analysis, in which nonsurvivors and nonresponders were not included, with probably higher frailty rates before ICU admission. In addition, the CFS is not validated for younger patients and despite the baseline questionnaire assessed frailty before hospital admission, it is also advocated that this has to be assessed for the last 2 weeks before hospitalization (45).

Pre-ICU anxiety and fatigue were included in the final model, meaning that already existing mental health problems are associated with a worse health status after ICU admission. The associations between pre-ICU variables and post-ICU outcomes were previously studied (3). Other research showed that ICU survivors encounter more diagnoses in primary care than matched reference patients after ICU stay and 12 months before ICU admission (46). Therefore, we believe that interventions to prevent and mitigate adverse outcomes should be focused on preventing worsening of existing health problems and developing new problems post-ICU. Thereby, most previously developed prediction models were used for screening patients after ICU discharge. This could be less useful because early identification (within 48 hr after ICU admission) is necessary for implementing early preventive interventions and informing and educating patients and their relatives at an early stage. The U.K. NICE guidelines also recommend to set rehabilitation goals for patients at risk before ICU discharge (6).

The PROSPECT model is currently being used in a multicenter clinical trial to select high-risk patients for evaluation of post-ICU care since February 2022 (47). Variables such as complications (e.g., delirium or ICU-acquired weakness) and serious deterioration of patients were not included in the model but can still be important risk factors for post-ICU health problems. Therefore, it remains important for ICU clinicians to stay alert for the occurrence of these uncontemplated events to provide these patients with the post-ICU care they need.

Some limitations need to be addressed. First, patients with an unplanned ICU admission completed the questionnaire after ICU admission and had to recall their health status before admission, which possibly led to an overestimation of baseline functioning. However, pre-ICU health status appeared to be the most important variable associated with post-ICU health problems in previous research (3). In addition, the medical and emergency surgery patients might be underrepresented, and therefore, the study sample’s results might be slightly better than ICU survivors in general. However, the percentage of elective surgical (planned surgery) patients corresponds to national percentages (48–51) and redevelopment of the model without elective surgical patients did not result in better model performance and different variable selection (Appendix 12, http://links.lww.com/CCM/H432). Second, the model was developed using actual length of ICU stay of historical patients. This means clinicians should use expected length of ICU stay of greater than or equal to 2 days or less than 2 days in case of early usage of the prediction model. Third, the prevalence of post-ICU health problems in our study population can be considered high but corresponds with previous studies (52). Fourth, specific data from patient’s electronic health records (EHRs), such as medication, was not available for the model development. However, adding more EHR data did not lead to better performance of priorly developed models predicting QoL and mortality (53, 54). Future research with possible new modeling techniques and more widespread sample sizes should show whether adding more data to the model improves predictive power.

## CONCLUSIONS

In this study, the PROSPECT model was developed and externally validated with distinctive predictive performance and good calibration. This prediction model can be used early after ICU admission for identifying patients with an increased risk of post-ICU problems 3 months after ICU admission.

## ACKNOWLEDGMENTS

We thank all the patients and their relatives for participating in this study. We also thank (in alphabetical order) Ed van Mackelenberg, Juliette Cruijsberg, Nicky Eijkenboom-Wattel, Marcel Houwer, Rachel Quibell-Melssen, Sanne Schröduer, and Sjef van der Velde for their advice and support in performing this study. Furthermore, we thank the National Foundation Family and Patient Centered Intensive Care and patient organization IC Connect for ICU survivors and their family members for their close cooperation.

## Supplementary Material
