# Supplementary material for: Prediction of Long-Term Physical, Mental, and Cognitive Problems Following Critical Illness: Development and External Validation of the PROSPECT Prediction Model*
Source: Crit Care Med. 2023 Dec 15;52(2):200–9. doi: 10.1097/CCM.0000000000006073 (PMC10793772; doi:10.1097/CCM.0000000000006073)
Supplement: Supplementary file 1 [file ccm-52-0200-s001.pdf]

Prediction of long term physical, mental and cognitive problems following critical illness: development and external validation of the PROSPECT model; van Sleeuwen et al.; Radboud University Medical Center; Dries.vansleeuwen@radboudumc.nl

Supplementary files

Content

Appendix 1. TRIPOD guidelines 3

Appendix 2. Definitions of candidate predictors......5

Appendix 3. All models......7

Description 8

e-Table S3a. Outline of developed models. 8

e-Table S3b. Legend of displayed model.......9

e-Table S3c. Model P3 (prediction model for physical problems; 3 months post ICU) 10

e-Table S3d. Model P12 (prediction model for physical problems; 12 months post ICU)......11

e-Table S3e. Model Pc (prediction model for physical problems; 3 + 12 months post ICU combined)......12

e-Table S3f. Model M3 (prediction model for mental problems; 3 months post ICU) 13

e-Table S3g. Model M12 (prediction model for mental problems; 12 months post ICU) 14

e-Table S3h. Model Mc (prediction model for mental problems; 3 + 12 months post ICU combined)......15

e-Table S3i. Model C3 (prediction model for cognitive problems; 3 months post ICU) 16

e-Table S3j. Model C12 (prediction model for cognitive problems; 12 months post ICU) 17

e-Table S3k. Model Cc (prediction model for cognitive problems; 3 + 12 months post ICU combined) 18

e-Table S3l. Model Gen3 (prediction model for general post-ICU health problems; 3 months post ICU) 19

e-Table S3m. Model Gen12 (prediction model for general post-ICU health problems; 12 months post ICU) 20

e-Table S3n. Model Genc (prediction model for general post-ICU health problems; 3 + 12 months post ICU combined)......21

Appendix 4. Missings......22

Appendix 5. Baseline characteristics of patients included in the analysis. 24
Appendix 6. Baseline characteristics datasets 27
Appendix 7. Baseline characteristics post-ICU health problems........ 30
Appendix 8. Formula 32
Appendix 9. Bootstrap samples........ 35
Appendix 10. Sensitivity and specificity plot of the PROSPECT model and cutoff points 36
Appendix 11. Predicted probabilities 37
Appendix 12. Model development and performance without elective surgical patients. 40

Appendix 1. TRIPOD guidelines

TRIPOD Checklist: Prediction Model Development and Validation

Section/Topic			Checklist Item	Page
**Title and abstract**				
Title	1	D;V	Identify the study as developing and/or validating a multivariable prediction model, the target population, and the outcome to be predicted.	1
Abstract	2	D;V	Provide a summary of objectives, study design, setting, participants, sample size, predictors, outcome, statistical analysis, results, and conclusions.	3
**Introduction**				
Background and objectives	3a	D;V	Explain the medical context (including whether diagnostic or prognostic) and rationale for developing or validating the multivariable prediction model, including references to existing models.	5
	3b	D;V	Specify the objectives, including whether the study describes the development or validation of the model or both.	5
**Methods**				
Source of data	4a	D;V	Describe the study design or source of data (e.g., randomized trial, cohort, or registry data), separately for the development and validation data sets, if applicable.	6
	4b	D;V	Specify the key study dates, including start of accrual; end of accrual; and, if applicable, end of follow-up.	6
Participants	5a	D;V	Specify key elements of the study setting (e.g., primary care, secondary care, general population) including number and location of centres.	6
	5b	D;V	Describe eligibility criteria for participants.	6
	5c	D;V	Give details of treatments received, if relevant.	6
Outcome	6a	D;V	Clearly define the outcome that is predicted by the prediction model, including how and when assessed.	6
	6b	D;V	Report any actions to blind assessment of the outcome to be predicted.	-
Predictors	7a	D;V	Clearly define all predictors used in developing or validating the multivariable prediction model, including how and when they were measured.	7
	7b	D;V	Report any actions to blind assessment of predictors for the outcome and other predictors.	-
Sample size	8	D;V	Explain how the study size was arrived at.	6
Missing data	9	D;V	Describe how missing data were handled (e.g., complete-case analysis, single imputation, multiple imputation) with details of any imputation method.	8
Statistical analysis methods	10a	D	Describe how predictors were handled in the analyses.	8
	10b	D	Specify type of model, all model-building procedures (including any predictor selection), and method for internal validation.	8
	10c	V	For validation, describe how the predictions were calculated.	8
	10d	D;V	Specify all measures used to assess model performance and, if relevant, to compare multiple models.	8
	10e	V	Describe any model updating (e.g., recalibration) arising from the validation, if done.	10

Risk groups	11	D;V	Provide details on how risk groups were created, if done.	-
Development vs. validation	12	V	For validation, identify any differences from the development data in setting, eligibility criteria, outcome, and predictors.	9
**Results**				
Participants	13a	D;V	Describe the flow of participants through the study, including the number of participants with and without the outcome and, if applicable, a summary of the follow-up time. A diagram may be helpful.	10
	13b	D;V	Describe the characteristics of the participants (basic demographics, clinical features, available predictors), including the number of participants with missing data for predictors and outcome.	9
	13c	V	For validation, show a comparison with the development data of the distribution of important variables (demographics, predictors and outcome).	10
Model development	14a	D	Specify the number of participants and outcome events in each analysis.	10
	14b	D	If done, report the unadjusted association between each candidate predictor and outcome.	-
Model specification	15a	D	Present the full prediction model to allow predictions for individuals (i.e., all regression coefficients, and model intercept or baseline survival at a given time point).	10
	15b	D	Explain how to the use the prediction model.	10
Model performance	16	D;V	Report performance measures (with CIs) for the prediction model.	10
Model-updating	17	V	If done, report the results from any model updating (i.e., model specification, model performance).	-
**Discussion**				
Limitations	18	D;V	Discuss any limitations of the study (such as nonrepresentative sample, few events per predictor, missing data).	12
Interpretation	19a	V	For validation, discuss the results with reference to performance in the development data, and any other validation data.	11
	19b	D;V	Give an overall interpretation of the results, considering objectives, limitations, results from similar studies, and other relevant evidence.	11
Implications	20	D;V	Discuss the potential clinical use of the model and implications for future research.	12
**Other information**				
Supplementary information	21	D;V	Provide information about the availability of supplementary resources, such as study protocol, Web calculator, and data sets.	Appendix
Funding	22	D;V	Give the source of funding and the role of the funders for the present study.	14

*Items relevant only to the development of a prediction model are denoted by D, items relating solely to a validation of a prediction model are denoted by V, and items relating to both are denoted D;V. We recommend using the TRIPOD Checklist in conjunction with the TRIPOD Explanation and Elaboration document.

Appendix 2. Definitions of candidate predictors

In total, 18 candidate predictors were selected based on results of previous MONITOR-IC research. This included age, gender, education level, quality of life before ICU using the EuroQol five-dimensional (EQ-5D-5L) questionnaire, symptoms of fatigue, anxiety, depression, and frailty before ICU admission, admission type (medical admission [nonsurgical admission, e.g. pneumonia], emergency surgical admission [acute surgical problem, e.g. spinal cord decompression], or elective surgical admission [planned surgery with ICU monitoring and/or treatment after surgery, e.g. coronary artery bypass grafting]), expected length of ICU stay of 2 or more days, severity of illness expressed in Acute Physiology and Chronic Health Evaluation (APACHE-IV score) 24 hours after ICU admission, and relevant comorbidities.

**Candidate predictors**	**Definition / categories / scale**
Education level	Low, defined as ≤ secondary school graduate<br>Middle<br>High, defined as postsecondary graduate
Admission type	Medical<br>Acute surgical<br>Planned surgical
Gender	Female or male.
Age	Years
Length of ICU stay (LOS)	The (expected) average number of 24 hour periods that a patient is treated in IC (treatment days). A 24 hour period is defined as attendance in IC.
Clinical Frailty Scale (CFS)	1 – 9
Checklist Individual Strength (CIS)	8 – 56
Cognitive Failures Questionnaire (CFQ)	0 – 100
Hospital Anxiety and Depression Scale: anxiety	0 – 21
Hospital Anxiety and Depression Scale: depression	0 – 21

Baseline EQ-5D-5L score	-0.446 – 1
Acquired Immune Deficiency Syndrome (AIDS)*	Defined as HIV-positive and clinical complications, or HIV-positive and CD4 < 200.
Immune insufficiency	Defined as prolonged immunosuppressive therapy, corticosteroid use, active chemotherapy or radiotherapy in past year, or documented humoral or cellular deficiencies
Hematologic malignancy	Defined as malignant lymphoma, acute leukaemia or multiple myeloma.
Cardiovascular insufficiency	Defined as New York Heart Association class IV.
Chronic Obstructive Pulmonary Disease (COPD)	Defined as chronic (> 6 months) use of bronchodilators or steroids for chronic lung disease.
Neoplasm	Defined as presence of confirmed metastasis or stage IV cancer.
Respiratory insufficiency	Defined as respiratory dependency or chronic lung disease resulting in severe movement restriction
Diabetes	Defined as medication dependent diabetes.
APACHE IV score	0 – 286

* Assigned to the immune insufficiency group.

Abbreviations: Intensive Care (IC); Intensive Care Unit (ICU); Quality of Life (QoL) measured with the EuroQol 5-Dimension 5-Level (EQ5D5L); Acute Physiology and Chronic Health Evaluation (APACHE score).

Appendix 3. All models

Table of contents

Description 8

e-Table S3a. Outline of developed models. 8

e-Table S3b. Legend of displayed model. 9

e-Table S3c. Model P3 (prediction model for physical problems; 3 months post ICU) 10

e-Table S3d. Model P12 (prediction model for physical problems; 12 months post ICU) 11

e-Table S3e. Model Pc (prediction model for physical problems; 3 + 12 months post ICU combined) 12

e-Table S3f. Model M3 (prediction model for mental problems; 3 months post ICU) 13

e-Table S3g. Model M12 (prediction model for mental problems; 12 months post ICU) 14

e-Table S3h. Model Mc (prediction model for mental problems; 3 + 12 months post ICU combined) 15

e-Table S3i. Model C3 (prediction model for cognitive problems; 3 months post ICU) 16

e-Table S3j. Model C12 (prediction model for cognitive problems; 12 months post ICU) 17

e-Table S3k. Model Cc (prediction model for cognitive problems; 3 + 12 months post ICU combined) 18

e-Table S3l. Model Gen3 (prediction model for general post-ICU health problems; 3 months post ICU) 19

e-Table S3m. Model Gen12 (prediction model for general post-ICU health problems; 12 months post ICU) 20

e-Table S3n. Model Genc (prediction model for general post-ICU health problems; 3 + 12 months post ICU combined) 21

Description

12 models were developed (table S3a). Each model is divided in subtypes depending on the number of added variables. A legend of the displayed models is shown in table S3b. Adding more predictors to the smallest model added improvement in terms of predictive performance. The less discriminating model (model C12 – predicting cognitive impairment 12 months post ICU. subtype 1 [table S3j]) had a C-statistics of 0.65 (95% CI: 0.62 to 0.69) and the best discriminating model (model M3 – predicting mental problems 3 months post ICU, subtype 7 [table S3f]) had a C-statistics of 0.79 (95% CI: 0.77 to 0.81).

e-Table S3a. Outline of developed models.

General post-ICU health problems were defined as symptoms in physical, mental, or cognitive domains.

Domain	Timeframe	Physical	Mental	Cognitive	General
	3 months	P3	M3	C3	Gen3
	12 months	P12	M12	C12	Gen12
	3 + 12 months	Pc	Mc	Cc	Genc

e-Table S3b. Legend of displayed model.

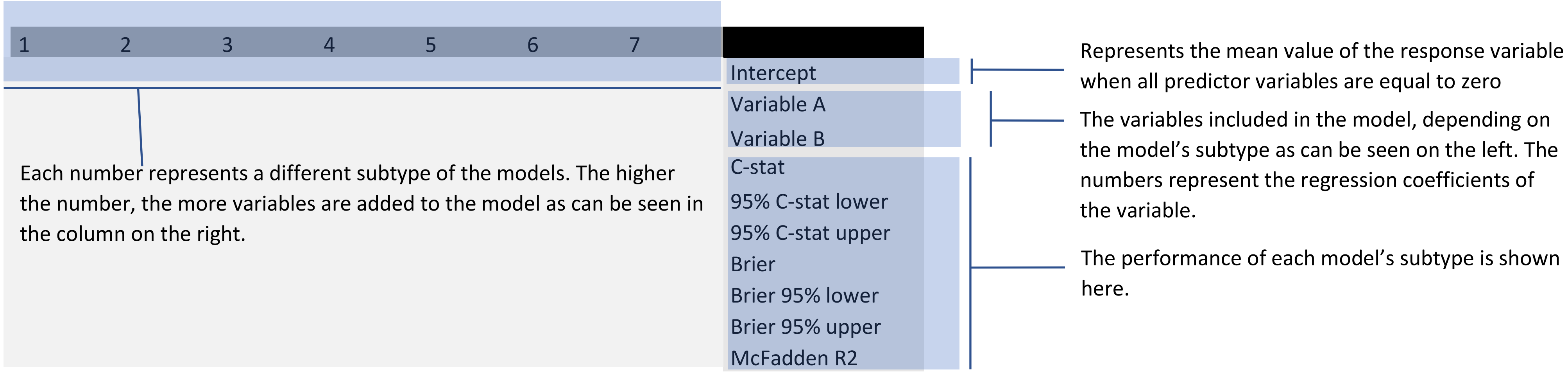

Abbreviations: Emergency surgical (EmS); Elective surgical (ElS); Length of ICU stay (LOS); Checklist individual Strength – fatigue subscale (CIS); Quality of Life (QoL) measured with the EuroQol 5-Dimension 5-Level (EQ5D5L); Hospital Anxiety and Depression Scale (HADS); Acute Physiology and Chronic Health Evaluation (APACHE score); Immune insuf. (Immune insufficiency); Hema. malign. (Hematologic malignancy); Cardiovasc. insuf. (Cardiovascular insufficiency); resp. insuf. (respiratory insufficiency); Chronic obstructive pulmonary disease (COPD).

Reference categories: low education [variable education]; medical Admission [variable Admission]

e-Table S3c. Model P3 (prediction model for physical problems; 3 months post ICU)

1	2	3	4	5	6	7	
-0.89218	-1.12656	-1.24573	-1.18372	-1.18838	-1.10658	-1.12654	Intercept
0.043159	0.044133	0.037429	0.037881	0.03653	0.040809	0.040186	CIS
0	0	0	0.324706	0.340903	0.325894	0.356179	Admission: EmS
0	0	0	-0.24652	-0.23976	-0.2546	-0.24808	Admission: ElS
0	0.557117	0.596646	0.479032	0.476397	0.476882	0.465933	LOS ≥ 2 days
0	0	0	0	0	-0.07218	-0.07442	Frailty
0	0	0.063404	0.070381	0.069721	0.069617	0.070379	HADS-anxiety
0	0	0	0	0	0	0.27362	Immune insuf.
0	0	0	0	0.472017	0.509553	0.514157	COPD
0.676991	0.690561	0.696987	0.70245	0.70734	0.709074	0.710335	C-stat
0.653946	0.670219	0.678211	0.681751	0.686532	0.690317	0.690682	95% C-stat lower
0.700601	0.713252	0.719754	0.725784	0.728998	0.734314	0.734597	95% C-stat upper
0.21835	0.21491	0.212956	0.210985	0.209848	0.209383	0.209031	Brier
0.211766	0.207933	0.205929	0.202779	0.201423	0.201822	0.199817	Brier 95% lower
0.224388	0.221414	0.218833	0.217466	0.215516	0.215684	0.215185	Brier 95% upper
0.068412	0.079953	0.088078	0.093865	0.096579	0.09798	0.099282	McFadden R2

e-Table S3d. Model P12 (prediction model for physical problems; 12 months post ICU)

1	2	3	4	5	6	7	8	9	10	
-1.42289	-1.20584	-1.34137	-0.45936	-0.42982	-1.01561	-1.21047	-1.21214	-1.21082	-0.93085	Intercept
0	0	0	-0.85541	-0.86464	-0.89725	-0.8805	-0.88365	-0.87477	-1.0684	QoL
0	0	0	0	0	0.01067	0.010846	0.010399	0.010205	0.010741	Age
0.042915	0.045229	0.0362	0.027894	0.029652	0.029313	0.029487	0.028771	0.028225	0.031588	CIS
0	0.076791	0.107809	0.137079	0.081254	0.089131	0.106837	0.143626	0.15016	0.133823	Admission: EmS
0	-0.66965	-0.7671	-0.71025	-0.77951	-0.80633	-0.69635	-0.69	-0.69125	-0.70121	Admission: ElS
0	0	0	0	0	0	0.285437	0.275465	0.275547	0.270861	LOS ≥ 2 days
0	0	0	0	0	0	0	0	0	-0.09139	Frailty
0	0	0.092093	0.082907	0.082417	0.081624	0.083064	0.084242	0.085026	0.083141	HADS-anxiety
0	0	0	0	0	0	0	0.337634	0.350693	0.358152	Immune insuf.
0	0	0	0	-1.19636	-1.17517	-1.15021	-1.32928	-1.37684	-1.38035	Hema. malign.
0	0	0	0	0	0	0	0	0.83029	0.888777	Cardiovasc. insuf.
0.675158	0.696466	0.711267	0.71766	0.722938	0.72728	0.731358	0.733214	0.734752	0.734978	C-stat
0.651125	0.674945	0.688918	0.696481	0.701579	0.705623	0.710723	0.712557	0.714046	0.715705	95% C-stat lower
0.70045	0.722309	0.735092	0.741996	0.747268	0.751829	0.756045	0.757166	0.758345	0.760663	95% C-stat upper
0.226358	0.220569	0.215492	0.213417	0.211739	0.210469	0.20954	0.208831	0.208198	0.207804	Brier
0.219064	0.212598	0.207632	0.204677	0.203192	0.201438	0.200326	0.199903	0.198532	0.197694	Brier 95% lower
0.232308	0.226763	0.221951	0.220152	0.218495	0.216619	0.215618	0.215501	0.214781	0.21389	Brier 95% upper
0.067972	0.087069	0.104329	0.110791	0.116548	0.120493	0.122949	0.124784	0.126628	0.128438	McFadden R2

e-Table S3e. Model Pc (prediction model for physical problems; 3 + 12 months post ICU combined)

1	2	3	4	5	6	7	8	9	10	11	12	
-1.12362	-0.88888	-1.08948	-0.84507	-1.0796	-1.08193	-0.59288	-0.56178	-0.58812	-0.28756	-0.09796	-0.08538	Intercept
0	-0.53804	0	-0.5589	-0.5594	-0.56076	-0.56083	-0.56121	-0.56146	-0.5621	-0.56322	-0.56299	TIME
0	0	0	0	0	0	-0.46743	-0.47786	-0.47851	-0.66736	-0.68197	-0.68432	QoL
0	0	0	0	0	0	0	0	0	0	-0.25986	-0.26128	Middle education
0	0	0	0	0	0	0	0	0	0	-0.13502	-0.14206	High education
0.042568	0.043045	0.036408	0.036928	0.037052	0.035853	0.031318	0.032129	0.03141	0.034692	0.035142	0.03497	CIS
0	0	0.199904	0.211632	0.231048	0.246765	0.258409	0.226502	0.260673	0.246636	0.252234	0.254244	Admission: EmS
0	0	-0.56474	-0.57766	-0.42845	-0.42178	-0.39413	-0.434	-0.43092	-0.43768	-0.4441	-0.44371	Admission: ElS
0	0	0	0	0.402939	0.398524	0.390169	0.382827	0.371181	0.366629	0.359433	0.359764	LOS ≥ 2 days
0	0	0	0	0	0	0	0	0	-0.08863	-0.0991	-0.10417	Frailty
0	0	0.078715	0.079294	0.081143	0.080182	0.074912	0.074587	0.075603	0.073494	0.07338	0.073453	HADS-anxiety
0	0	0	0	0	0	0	0	0.332971	0.340816	0.355878	0.362308	Immune insuf.
0	0	0	0	0	0	0	-0.68742	-0.84998	-0.84385	-0.84633	-0.8671	Hema. malign.
0	0	0	0	0	0	0	0	0	0	0	0.571119	Cardiovasc. insuf.
0	0	0	0	0	0.418632	0.406474	0.397795	0.399604	0.43591	0.41085	0.394952	COPD
0.674427	0.689046	0.699723	0.712177	0.716823	0.720216	0.721807	0.724295	0.72605	0.727777	0.729337	0.730267	C-stat
0.656465	0.670405	0.68289	0.695412	0.7001	0.705886	0.70524	0.708983	0.710911	0.712602	0.71296	0.716518	95% C-stat lower
0.692316	0.707372	0.719403	0.731281	0.735878	0.739304	0.741103	0.744204	0.744587	0.746444	0.749421	0.748374	95% C-stat upper
0.22573	0.222019	0.21831	0.214453	0.212978	0.212073	0.211554	0.210854	0.210263	0.209805	0.209259	0.208977	Brier
0.220304	0.21657	0.212166	0.207847	0.206561	0.205715	0.204982	0.204476	0.202841	0.20332	0.202466	0.201995	Brier 95% lower
0.231171	0.226812	0.223703	0.219585	0.217906	0.217263	0.21659	0.215731	0.21454	0.214557	0.214131	0.214063	Brier 95% upper
0.067106	0.07878	0.091877	0.104023	0.109053	0.111262	0.113103	0.114957	0.116769	0.118519	0.120238	0.121139	McFadden R2

e-Table S3f. Model M3 (prediction model for mental problems; 3 months post ICU)

1	2	3	4	5	6	7	
-1.68019	-1.60977	-1.85199	-1.51424	-1.21001	-1.39642	-1.03819	Intercept
0	0	0	-0.45892	-0.50476	-0.49995	-0.51642	Middle education
0	0	0	-0.47554	-0.5354	-0.55909	-0.57658	High education
0	0.57502	0.625719	0.628447	0.586311	0.601987	0.569331	Admission: EmS
0	-0.76749	-0.75421	-0.75057	-0.7889	-0.67007	-0.75274	Admission: ElS
0	0	0	0	0	0.331108	0.413589	LOS ≥ 2 days
0	0	0	0	-0.1184	-0.1197	-0.11974	Frailty
0.216152	0.247491	0.17797	0.178373	0.178826	0.181667	0.179075	HADS-anxiety
0	0	0.11952	0.116754	0.136321	0.135274	0.138759	HADS-depression
0	0	0	0	0	0	-0.0064	APACHE IV score
0.730008	0.758738	0.776515	0.781756	0.785423	0.787725	0.789312	C-stat
0.708961	0.740317	0.757949	0.763977	0.766566	0.769398	0.771742	95% C-stat lower
0.752378	0.780434	0.797705	0.803266	0.807643	0.808289	0.809487	95% C-stat upper
0.197368	0.18818	0.182501	0.180694	0.179628	0.179016	0.178399	Brier
0.189058	0.179469	0.173172	0.170434	0.170337	0.169357	0.16878	Brier 95% lower
0.205074	0.195615	0.189528	0.187601	0.186543	0.186122	0.185415	Brier 95% upper
0.120551	0.156122	0.177283	0.183461	0.187497	0.190551	0.192863	McFadden R2

e-Table S3g. Model M12 (prediction model for mental problems; 12 months post ICU)

1	2	3	4	5	6	7	
-1.85195	-1.71939	-1.89639	-1.53769	-1.56018	-1.32602	-1.46808	Intercept
0	0	0	-0.3924	-0.39119	-0.42621	-0.4271	Middle education
0	0	0	-0.57314	-0.57759	-0.62389	-0.64699	High education
0	0.254744	0.276495	0.262042	0.27317	0.236083	0.249799	Admission: EmS
0	-0.69415	-0.70118	-0.6983	-0.69738	-0.73038	-0.62716	Admission: ElS
0	0	0	0	0	0	0.262554	LOS ≥ 2 days
0	0	0	0	0	-0.08786	-0.08945	Frailty
0.209576	0.231142	0.1681	0.164801	0.164916	0.163589	0.164987	HADS-anxiety
0	0	0.104201	0.10203	0.101942	0.117305	0.116695	HADS-depression
0	0	0	0	0.909179	1.036442	1.040228	Cardiovasc. insuf.
0.726381	0.746961	0.761732	0.766386	0.767508	0.768774	0.770701	C-stat
0.703459	0.725013	0.740738	0.744877	0.748299	0.74752	0.752112	95% C-stat lower
0.750453	0.771741	0.784927	0.790383	0.791518	0.794074	0.796352	95% C-stat upper
0.188406	0.18335	0.179299	0.177467	0.176755	0.176324	0.175829	Brier
0.179336	0.173357	0.169206	0.167108	0.16654	0.165979	0.165288	Brier 95% lower
0.197709	0.191925	0.187168	0.185267	0.185295	0.184345	0.183777	Brier 95% upper
0.112619	0.134133	0.150132	0.15688	0.15944	0.161586	0.163521	McFadden R2

e-Table S3h. Model Mc (prediction model for mental problems; 3 + 12 months post ICU combined)

1	2	3	4	5	6	7	8	9	
-1.75775	-1.65855	-1.87056	-1.52169	-1.26419	-1.42973	-1.33639	-1.32756	-1.29655	Intercept
0	0	0	0	0	0	-0.21279	-0.21113	-0.21304	TIME
0	0	0	-0.4287	-0.46734	-0.46516	-0.46392	-0.46623	-0.47726	Middle education
0	0	0	-0.52511	-0.57525	-0.59907	-0.59287	-0.59986	-0.60822	High education
0	0.422235	0.458563	0.453627	0.415404	0.429752	0.433794	0.437559	0.431725	Admission: EmS
0	-0.73016	-0.72686	-0.72342	-0.75729	-0.64549	-0.64999	-0.65021	-0.62914	Admission: ElS
0	0	0	0	0	0.299992	0.298663	0.298917	0.283619	LOS ≥ 2 days
0	0	0	0	-0.09791	-0.09945	-0.09884	-0.10768	-0.10909	Frailty
0.213094	0.239512	0.172679	0.171392	0.170916	0.173032	0.173593	0.172918	0.173972	HADS-anxiety
0	0	0.112858	0.110212	0.126792	0.125968	0.125487	0.126905	0.126558	HADS-depression
0	0	0	0	0	0	0	0	-0.30548	neoplasm
0	0	0	0	0	0	0	0.6439	0.644964	Cardiovasc. insuf.
0.728323	0.752608	0.769368	0.774699	0.776834	0.779197	0.779868	0.780574	0.781399	C-stat
0.708725	0.736101	0.752439	0.75903	0.760013	0.763473	0.76294	0.765829	0.76546	95% C-stat lower
0.747225	0.772234	0.78695	0.791919	0.794114	0.796995	0.798896	0.800226	0.801191	95% C-stat upper
0.193669	0.186634	0.181659	0.179847	0.179208	0.178608	0.178201	0.177941	0.177549	Brier
0.185811	0.179295	0.173535	0.171707	0.170932	0.170228	0.17054	0.170273	0.168822	Brier 95% lower
0.200074	0.193848	0.188372	0.186239	0.186011	0.185479	0.184616	0.184195	0.183381	Brier 95% upper
0.117019	0.145233	0.164117	0.17047	0.173226	0.175759	0.177295	0.17855	0.179433	McFadden R2

e-Table S3i. Model C3 (prediction model for cognitive problems; 3 months post ICU)

1	2	3	4	5	6	7	
-2.81378	-2.55309	-2.82407	-2.52695	-2.23318	-2.23654	-2.46215	Intercept
0	0	0	0	0	0	0.015239	CIS
0	-0.01386	0.004248	-0.00947	-0.06555	-0.05019	-0.042	Admission: EmS
0	-0.95282	-0.77015	-0.75538	-0.808	-0.81183	-0.80155	Admission: ElS
0	0	0.45089	0.473671	0.49296	0.494367	0.496732	LOS ≥ 2 days
0	0	0	0	-0.1082	-0.12546	-0.19257	Frailty
0	0	0	-0.44289	-0.48698	-0.50778	-0.50298	Male sex
0.121759	0.14352	0.142688	0.132141	0.143767	0.143107	0.128976	HADS-anxiety
0	0	0	0	0	0.558974	0.555076	diabetes
0.658531	0.692008	0.699284	0.711337	0.710107	0.709993	0.716759	C-stat
0.621148	0.657301	0.666724	0.680285	0.681276	0.680211	0.688665	95% C-stat lower
0.699688	0.731374	0.736185	0.74988	0.75085	0.753145	0.757357	95% C-stat upper
0.093761	0.091961	0.09138	0.091054	0.09042	0.089758	0.089597	Brier
0.082478	0.080939	0.080782	0.080626	0.080101	0.078863	0.079072	Brier 95% lower
0.103378	0.102082	0.100554	0.099983	0.099996	0.099833	0.099128	Brier 95% upper
0.040719	0.068102	0.07389	0.080032	0.083953	0.088305	0.092256	McFadden R2

e-Table S3j. Model C12 (prediction model for cognitive problems; 12 months post ICU)

1	2	3	4	5	6	7	8	9	
-2.74218	-2.57804	-1.82848	-2.23159	-2.20569	-2.46819	-1.88779	-1.88604	-2.15697	Intercept
0	0	-0.01344	-0.01343	-0.01281	0	-0.01106	-0.01033	-0.01311	Age
0	0	0	0.015585	0.017532	0.026428	0.02564	0.025661	0.024596	CIS
0	0.186415	0.178089	0.221796	0.140048	0.064713	0.069246	0.068398	0.091677	Admission: EmS
0	-0.75915	-0.71665	-0.68444	-0.73365	-0.82852	-0.78896	-0.74783	-0.61521	Admission: ElS
0	0	0	0	0	-0.15934	-0.14923	-0.15259	-0.14754	Frailty
0	0	0	0	0	-0.39572	-0.35821	-0.38417	-0.4072	Male sex
0.129586	0.147647	0.149887	0.126733	0.123721	0.118055	0.120665	0.120602	0.123373	HADS-anxiety
0	0	0	0	0	0	0	-0.68282	-0.70843	neoplasm
0	0	0	0	-0.63651	-0.67471	-0.64122	-0.5934	-0.63441	Immune insuf.
0	0	0	0	0	0	0	0	0.007423	APACHE IV score
0.6547	0.694594	0.703212	0.715241	0.724472	0.724136	0.726153	0.728005	0.73008	C-stat
0.61562	0.660059	0.66928	0.681576	0.692407	0.696007	0.699356	0.700273	0.70446	95% C-stat lower
0.692947	0.737384	0.739745	0.750248	0.763132	0.760611	0.764192	0.767853	0.773302	95% C-stat upper
0.101166	0.100028	0.099552	0.099446	0.099459	0.099029	0.098481	0.098023	0.097553	Brier
0.089711	0.088612	0.089176	0.088625	0.086701	0.087594	0.086977	0.08675	0.085709	Brier 95% lower
0.111661	0.110384	0.109821	0.108426	0.109073	0.108723	0.108381	0.107272	0.106465	Brier 95% upper
0.044547	0.066067	0.072063	0.077668	0.08278	0.087334	0.091217	0.094241	0.097225	McFadden R2

e-Table S3k. Model Cc (prediction model for cognitive problems; 3 + 12 months post ICU combined)

1	2	3	4	5	6	7	8	9	10	11	
-2.77709	-2.56297	-2.29214	-2.52326	-2.36407	-2.58677	-2.5475	-2.50468	-2.53839	-2.16798	-2.22224	Intercept
0	0	0	0	0	0	0	0	0	0	0.110727	TIME
0	0	0	0	0	0	0	0	0	-0.007	-0.00705	Age
0	0	0	0	0.019902	0.019935	0.021322	0.021374	0.021493	0.021038	0.02099	CIS
0	0.089127	0.075999	0.090158	0.043165	0.054662	-0.00502	-0.00512	-0.0344	-0.03934	-0.04167	Admission: EmS
0	-0.85792	-0.85583	-0.68548	-0.88819	-0.71499	-0.74073	-0.71317	-0.73045	-0.7083	-0.70687	Admission: ElS
0	0	0	0.409067	0	0.425034	0.432537	0.403907	0.425338	0.426249	0.427362	LOS ≥ 2 days
0	0	0	0	-0.16614	-0.1726	-0.1676	-0.16999	-0.15317	-0.14565	-0.14482	Frailty
0	0	-0.39252	-0.40827	-0.41671	-0.43737	-0.43983	-0.45336	-0.45589	-0.43211	-0.43122	Male sex
0.125346	0.14518	0.136637	0.135364	0.125662	0.125055	0.122379	0.12282	0.123491	0.124281	0.124395	HADS-anxiety
0	0	0	0	0	0	0	-0.55884	-0.56536	-0.53124	-0.52998	neoplasm
0	0	0	0	0	0	-0.5287	-0.48763	-0.4755	-0.45558	-0.45571	Immune insuf.
0	0	0	0	0	0	0	0	-0.80913	-0.86218	-0.87038	Resp. insuf.
0.656513	0.693596	0.702635	0.710895	0.710393	0.718299	0.723761	0.725198	0.725218	0.726942	0.727399	C-stat
0.625593	0.665003	0.673183	0.686198	0.686549	0.693263	0.697599	0.703298	0.702417	0.705518	0.705088	95% C-stat lower
0.686793	0.724823	0.732643	0.744229	0.743769	0.749217	0.754695	0.7566	0.761571	0.764311	0.763867	95% C-stat upper
0.097397	0.095924	0.09573	0.095384	0.094985	0.094504	0.094381	0.094044	0.093732	0.093574	0.093529	Brier
0.087941	0.08748	0.086871	0.086461	0.0858	0.085716	0.084922	0.084821	0.084174	0.084459	0.08437	Brier 95% lower
0.10536	0.104763	0.104476	0.103933	0.103311	0.102525	0.10271	0.102026	0.101497	0.101559	0.101453	Brier 95% upper
0.042468	0.066737	0.071701	0.076416	0.080515	0.085556	0.089257	0.09136	0.093372	0.094961	0.095362	McFadden R2

e-Table S3l. Model Gen3 (prediction model for general post-ICU health problems; 3 months post ICU)

1	2	3	4	5	6	7	8	9	10	
-0.48774	-0.69691	-0.62793	-0.85484	-0.86043	-0.82387	-0.69245	-0.41728	-0.14908	-0.13333	Intercept
0	0	0	0	0	0	0	-0.37892	-0.39267	-0.4059	Middle education
0	0	0	0	0	0	0	-0.25179	-0.25644	-0.26527	High education
0.044887	0.032901	0.03406	0.034185	0.032472	0.027093	0.033007	0.034018	0.034445	0.033788	CIS
0	0	0.404439	0.420334	0.438867	0.443866	0.423147	0.424655	0.389152	0.418608	Admission: EmS
0	0	-0.47742	-0.34294	-0.33547	-0.33816	-0.35995	-0.36999	-0.43642	-0.43815	Admission: ElS
0	0	0	0.393188	0.39217	0.388462	0.385975	0.374646	0.434458	0.427027	LOS ≥ 2 days
0	0	0	0	0	0	-0.11093	-0.12728	-0.12839	-0.13169	Frailty
0	0.124691	0.138508	0.141216	0.140676	0.116543	0.113969	0.114697	0.112413	0.113614	HADS-anxiety
0	0	0	0	0	0.05562	0.061854	0.06055	0.062006	0.061778	HADS-depression
0	0	0	0	0	0	0	0	0	0.319454	Immune insuf.
0	0	0	0	0.621616	0.592714	0.647541	0.627458	0.647801	0.654814	COPD
0	0	0	0	0	0	0	0	-0.00474	-0.00524	APACHE IV score
0.682838	0.713236	0.726705	0.729665	0.734298	0.737197	0.740516	0.743609	0.745696	0.747636	C-stat
0.656791	0.690747	0.703719	0.707657	0.713379	0.715805	0.719303	0.722805	0.726604	0.730177	95% C-stat lower
0.70805	0.738673	0.751568	0.753947	0.760286	0.761543	0.766483	0.766926	0.772703	0.772938	95% C-stat upper
0.192853	0.187052	0.182685	0.181421	0.180469	0.179717	0.178979	0.178046	0.177599	0.177269	Brier
0.184675	0.178559	0.173333	0.172676	0.170959	0.170635	0.169584	0.1675	0.167756	0.167427	Brier 95% lower
0.200005	0.194568	0.190071	0.188777	0.187633	0.187586	0.186452	0.185122	0.184692	0.184106	Brier 95% upper
0.071345	0.098906	0.114491	0.119138	0.12302	0.126005	0.12906	0.132428	0.133835	0.135422	McFadden R2

e-Table S3m. Model Gen12 (prediction model for general post-ICU health problems; 12 months post ICU)

1	2	3	4	5	6	7	
-1.051	-1.22478	-1.04244	-0.63811	0.096338	0.115152	0.075689	Intercept
0	0	0	0	-0.71823	-0.72934	-0.71419	QoL
0	0	0	-0.48281	-0.47817	-0.47207	-0.4479	Middle education
0	0	0	-0.52774	-0.50353	-0.4879	-0.46269	High education
0.04244	0.032053	0.033692	0.033279	0.026435	0.027669	0.026547	CIS
0	0	0.233961	0.221386	0.239991	0.203266	0.21817	Admission: EmS
0	0	-0.79979	-0.80003	-0.75841	-0.80915	-0.80008	Admission: ElS
0	0.103019	0.122688	0.119424	0.111842	0.111448	0.110314	HADS-anxiety
0	0	0	0	0	-0.8303	-0.81355	Hema. malign.
0	0	0	0	0	0	0.459267	COPD
0.673153	0.697282	0.724585	0.731917	0.736119	0.738826	0.741515	C-stat
0.647897	0.674764	0.702322	0.712025	0.714299	0.717005	0.718989	95% C-stat lower
0.698807	0.721549	0.748672	0.756421	0.761816	0.765627	0.766054	95% C-stat upper
0.224764	0.218191	0.209164	0.207031	0.205829	0.205011	0.204127	Brier
0.217059	0.210678	0.200846	0.198365	0.195648	0.194919	0.194689	Brier 95% lower
0.230974	0.224666	0.216685	0.213375	0.213207	0.211664	0.211092	Brier 95% upper
0.065961	0.086989	0.116149	0.123258	0.127248	0.129957	0.132377	McFadden R2

e-Table S3n. Model Genc (prediction model for general post-ICU health problems; 3 + 12 months post ICU combined)

1	2	3	4	5	6	7	8	9	10	11	12	13	14	
-0.74142	-0.92993	-0.80157	-0.49747	-0.18064	-0.20428	-0.37298	-0.3497	-0.2246	0.30776	0.324884	0.318538	0.330588	0.548471	Intercept
0	0	0	-0.67191	-0.66996	-0.67204	-0.67025	-0.66765	-0.66791	-0.66781	-0.66867	-0.6694	-0.66927	-0.67075	TIME
0	0	0	0	0	0	0	0	0	-0.47352	-0.47909	-0.48232	-0.48256	-0.48137	QoL
0	0	0	0	-0.42383	-0.40083	-0.39198	-0.38672	-0.41852	-0.42594	-0.4248	-0.43686	-0.43903	-0.4495	Middle education
0	0	0	0	-0.37477	-0.34957	-0.35859	-0.34094	-0.37946	-0.38038	-0.37086	-0.37833	-0.38572	-0.39047	High education
0.043053	0.032016	0.033278	0.033843	0.033545	0.032111	0.032118	0.028244	0.032425	0.030003	0.030675	0.030168	0.029886	0.030086	CIS
0	0	0.304014	0.319304	0.318829	0.335459	0.348251	0.348255	0.332112	0.33606	0.3128	0.340894	0.341683	0.317727	Admission: EmS
0	0	-0.61731	-0.636	-0.63608	-0.62717	-0.52213	-0.52925	-0.54485	-0.52582	-0.55839	-0.55872	-0.55849	-0.60966	Admission: ElS
0	0	0	0	0	0	0.289259	0.284919	0.283348	0.274841	0.267463	0.253733	0.254415	0.303216	LOS ≥ 2 days
0	0	0	0	0	0	0	0	-0.08119	-0.11231	-0.11086	-0.11317	-0.11784	-0.1188	Frailty
0	0.11187	0.127962	0.130058	0.12859	0.127611	0.129352	0.111509	0.109384	0.105604	0.105793	0.107079	0.106749	0.105262	HADS-anxiety
0	0	0	0	0	0	0	0.040094	0.044966	0.042061	0.041429	0.040767	0.041726	0.042683	HADS-depression
0	0	0	0	0	0	0	0	0	0	0	0.295093	0.301193	0.322588	Immune insuf.
0	0	0	0	0	0	0	0	0	0	-0.54883	-0.68807	-0.70338	-0.67023	Hema. malign.
0	0	0	0	0	0	0	0	0	0	0	0	0.5444	0.607923	Cardiovasc. insuf.
0	0	0	0	0	0.538383	0.530553	0.510849	0.542265	0.546553	0.538547	0.540323	0.523462	0.536787	COPD
0	0	0	0	0	0	0	0	0	0	0	0	0	-0.00393	APACHE IV score
0.675836	0.702672	0.721289	0.73531	0.740141	0.743231	0.745291	0.747038	0.748014	0.749065	0.750448	0.751807	0.752434	0.753785	C-stat
0.655651	0.683473	0.702523	0.718224	0.724719	0.726894	0.728863	0.732181	0.73192	0.733556	0.736625	0.738719	0.736902	0.739111	95% C-stat lower
0.695065	0.722384	0.740553	0.753856	0.758364	0.762942	0.764036	0.766263	0.767161	0.769696	0.769212	0.772991	0.773617	0.773549	95% C-stat upper
0.212199	0.20576	0.199716	0.195178	0.19371	0.192899	0.19203	0.191572	0.191304	0.190803	0.190399	0.190129	0.190001	0.189639	Brier
0.206007	0.19909	0.192137	0.188012	0.186634	0.186254	0.18404	0.18392	0.183755	0.183093	0.18272	0.181711	0.181174	0.181715	Brier 95% lower
0.218025	0.211993	0.206119	0.200955	0.199834	0.19928	0.197735	0.197412	0.196975	0.197136	0.196442	0.195302	0.195336	0.195444	Brier 95% upper
0.067259	0.090986	0.111223	0.128106	0.13272	0.135812	0.138267	0.139863	0.141414	0.142835	0.143951	0.145279	0.146014	0.146916	McFadden R2

Appendix 4. Missings

Number of missing values per candidate predictor in development dataset (n = 1454)

Variable	Missing values (N)	Percentage of missing values (%)
Education level	23	1.58
Admission type	0	-
Gender	0	-
Age	0	-
LOS	0	-
Frailty	7	0.48
HADS-anxiety	10	0.69
HADS-depression	7	0.48
QoL	40	2.75
CIS	18	1.24
Immune insufficiency	0	-
Hematologic malignancy	0	-

Cardiovascular insufficiency	0	-
COPD	0	-
Neoplasm	0	-
Respiratory insufficiency	0	-
Diabetes	0	-
APACHE IV score	0	-

Abbreviations: Length of ICU stay (LOS); Checklist individual Strength – fatigue subscale (CIS); Quality of Life (QoL) measured with the EuroQol 5-Dimension 5-Level (EQ5D5L); Hospital Anxiety and Depression Scale (HADS); Acute Physiology and Chronic Health Evaluation (APACHE score), Chronic obstructive pulmonary disease (COPD).

Appendix 5. Baseline characteristics of patients included in the analysis.

Variable	Categories	Development (N = 1454)	Validation (N = 1022)	P value	All patients (N = 2476)
Age, years – mean (SD)		58.78 (15.94)	64.46 (12.77)	<.001	61.02 (15.02)
	Missing	-	72		72
Gender – n/N (%)	Male	898 (61.76)	579 (56.65))	.69	1477 (59.65)
	Female	556 (38.24)	371 (36.30)		927 (37.44)
	Missing	-	72 (7.05)		72 (2.91)
Education level – n/N (%)	Low	409 (28.13)	372 (36.40)	<.001	781 (31.54)
	Middle	660 (45.39)	425 (41.59)		1085 (43.82)
	High	362(24.90)	212 (20.74)		574 (23.18)
	Missing	23 (1.58)	13 (1.27)		36 (1.45)
Admission type – n/N (%)	Medical	556 (38.24)	646 (63.21)	<.001	1202 (48.55)
	Emergency surgery	244 (16.78)	129 (12.62)		373 (15.06)
	Elective surgery	654 (44.98)	170 (16.63)		824 (33.28)
	Missing	-	77 (7.53)		77 (3.11)
Length of ICU stay – n/N (%)	<2 days	888 (61.07)	509 (49.80)	<.001	1397 (56.42)
	≥ 2 days	566 (38.93)	441 (43.15)		1007 (40.67)
	Missing	-	72 (7.05)		72 (2.91)
APACHE score– mean (SD)		53.70 (23.01)	58.68 (25.87)	<.001	55.67 (24.30)
	Missing	-	72 (7.05)		72 (2.91)
Comorbidities – n/N (%)	Chronic renal insufficiency	59 (4.06)	48 (4.70)	.25	107 (4.32)
	Chronic dialysis	19 (1.31)	7 (0.68)	.19	26 (1.05)
	Neoplasm	129 (8.87)	36 (3.52)	<.001	165 (6.66)
	Immune insufficiency	209 (14.37)	94 (9.20)	<.001	303 (12.24)
	Hematologic malignancy	40 (2.75)	13 (1.27)	.02	53 (2.14)
	Cirrhosis	6 (0.41)	5 (0.49)	.76	11 (0.44)
	Cardiovascular insufficiency	30 (2.06)	19 (1.86)	.92	49 (1.98)
	Respiratory insufficiency	37 (2.54)	50 (4.89)	<.001	87 (3.51)
	COPD	148 (10.18)	150 (14.68)	<.001	298 (12.04)
	Diabetes	148 (10.18)	131 (12.82)	.01	279 (11.27)
	Missing	-	72 (7.05)		72 (2.91)
CPR – n/N (%)		68 (4.68)	57 (5.58)	.15	125 (5.05)
	Missing	-	72 (7.05)		72 (2.91)
Mechanical ventilation[₭] – n/N (%)		907 (62.38)	355 (34.74)	<.001	1262 (50.97)
	Missing	-	72 (7.05)		72 (2.91)
Frailty (CFS)[₱] – n/N (%)	Frail	218 (14.99)	162 (15.85)	.01	380 (15.35)
	Not frail	1229 (84.53)	849 (83.07)		2078 (83.93)
	Missing	7 (0.48)	11 (1.08)		18 (0.73)
Fatigue (CIS)[₫] – n/N (%)	Fatigue	857 (58.94)	664 (64.97)	<.001	1521 (61.43)
	No fatigue	579 (39.82)	351 (34.34)		930 (37.56)
	Missing	18 (1.24)	7 (0.68)		25 (1.00)
HADS-Anxiety[€] – n/N (%)	Anxiety symptoms	361 (24.3)	271 (26.52)	.38	632 (25.53)
	No anxiety	1083 (74.48)	749 (73.29)		1832 (74.00)
	Missing	10 (0.69)	2 (0.20)		12 (0.48)
HADS-Depression[£] – n/N (%)	Depression symptoms	351 (24.14)	278 (27.20)	.09	629 (25.40)
	No depression	1096 (75.38)	742 (72.60)		1838 (74.23)

	Missing	7 (0.48)	2 (0.20)		9 (0.36)
Cognitive impairment¥ – n/N (%)	Yes	72 (4.95)	74 (7.24)	.23	146 (5.90)
	No	1073 (73.80)	898 (87.87)		1971 (79.60)
	Missing	309 (21.25)	50 (4.89)		359 (14.50)
QoL (EQ-5D-5L) – median [IQR]		0.81 [0.55-0.89]	0.77 [0.53-0.89]	.02	0.79 [0.54-0.89]
	Missing	40	16		56

₱ Positive score defined by a score of ≥5 on the Clinical Frailty Scale (CFS)

₫ Positive score defined by a score of >37 on the Checklist Individual Strength – fatigue subscale (CIS-8)

€ Positive score defined by a score of ≥ 8 on the Hospital Anxiety and Depression Scale (HADS) anxiety subscales

£ Positive score defined by a score of ≥ 8 on the Hospital Anxiety and Depression Scale (HADS) depression subscales

¥ Positive score defined as cognitive impairment with a score of ≥43 on the abbreviated Cognitive Failure Questionnaire (CFQ-14)

₭ Mechanical ventilation within first 24 hours of ICU admission

Abbreviation: APACHE, Acute Physiology and Chronic Health Evaluation; ICU, Intensive Care Unit; IQR, Interquartile range; COPD, chronic obstructive pulmonary disease; CPR, cardiopulmonary resuscitation; SD, standard deviation.

Appendix 6. Baseline characteristics datasets

Baseline characteristics of patients included in the development and external validation dataset of the PROSPECT model (n = 1244 + 864 = 2108), and patients with insufficient baseline or outcome data for the development and external validation dataset of the PROSPECT model (n = 210 + 158 = 368).

Variable	Categories	Included in development and external validation PROSPECT model (N = 2108)	Insufficient baseline and outcome data for PROSPECT model (N = 368)	P value
Age, years – mean (SD)		61.29 (14.70)	59.08 (17.05)	.03
	Missing	-	72 (19.57)	
Gender – n/N (%)	Male	1288 (61.10)	189 (51.36)	.40
	Female	820 (38.90)	107 (29.08)	
	Missing	-	72 (19.57)	
Education level – n/N (%)	Low	660 (31.31)	121 (32.88)	.02
	Middle	925 (43.88)	160 (43.48)	
	High	514 (24.38)	60 (16.30)	
	Missing	9 (0.43)	27 (7.33)	
Admission type – n/N (%)	Medical	1061 (50.33)	141 (38.31)	.69
	Emergency surgery	323 (15.32)	50 (13.59)	
	Elective surgery	724 (34.35)	100 (27.17)	
	Missing	-	77 (20.92)	
Length of ICU stay – n/N (%)	<2 days	1225 (58.11)	172 (46.74)	1.0
	≥ 2 days	883 (41.89)	124 (33.70)	
	Missing	-	72 (19.57)	
APACHE score– mean (SD)		55.65 (24.20)	55.81 (25.06)	.92
	Missing	-	72	
Comorbidities – n/N (%)	Chronic renal insufficiency	94 (4.46)	13 (3.53)	1.0
	Chronic dialysis	22 (1.04)	4 (1.09)	.86
	Neoplasm	142 (6.74)	23 (6.25)	.59
	Immune insufficiency	261 (12.38)	42 (11.41)	.43
	Hematologic malignancy	47 (2.22)	6 (1.63)	1.0
	Cirrhosis	9 (0.43)	2 (0.54)	.64
	Cardiovascular insufficiency	45 (2.13)	4 (1.09)	.50
	Respiratory insufficiency	78 (3.70)	9 (2.45)	.69
	COPD	265 (12.57)	33 (8.97)	.55
	Diabetes	240 (11.39)	39 (10.60)	.42
	Missing	-	72 (19.57)	
CPR – n/N (%)		105 (4.98)	20 (5.43)	.25

	Missing	-	72 (19.57)	
Frailty (CFS)[₱] – n/N (%)	Frail	319 (15.13)	61 (16.58)	.43
	Not frail	1780 (84.44)	298 (80.98)	
	Missing	9 (0.43)	9 (2.45)	
Fatigue (CIS)[₫] – n/N (%)	Fatigue	1311 (62.19)	210 (57.07)	.78
	No fatigue	797 (37.81)	133 (36.14)	
	Missing	-	25 (6.79)	
HADS-Anxiety[€] – n/N (%)	Anxiety symptoms	546 (25.90)	86 (23.37)	.53
	No anxiety	1562 (74.10)	270 (73.37)	
	Missing	-	12 (3.26)	
HADS-Depression[£] – n/N (%)	Depression symptoms	531 (25.19)	98 (26.63)	.43
	No depression	1577 (74.81)	261 (70.92)	
	Missing	-	9 (2.45)	
Cognitive impairment[¥] – n/N (%)	Yes	127 (6.02)	19 (5.16)	.75
	No	1739 (82.50)	232 (63.04)	
	Missing	242 (11.48)	117 (31.79)	
QoL (EQ-5D-5L) – median [IQR]		0.79 [0.55-0.89]	0.74 [0.48-0.89]	<.001
	Missing	11 (0.52)	45 (12.23)	

₱ Positive score defined by a score of ≥5 on the Clinical Frailty Scale (CFS)
₫ Positive score defined by a score of >37 on the Checklist Individual Strength – fatigue subscale (CIS-8)
€ Positive score defined by a score of ≥ 8 on the Hospital Anxiety and Depression Scale (HADS) anxiety subscales
£ Positive score defined by a score of ≥ 8 on the Hospital Anxiety and Depression Scale (HADS) depression subscales
¥ Positive score defined as cognitive impairment with a score of ≥43 on the abbreviated Cognitive Failure Questionnaire (CFQ-14)

Abbreviations: APACHE, Acute Physiology and Chronic Health Evaluation; ICU, Intensive Care Unit; IQR, Interquartile range; COPD, chronic obstructive pulmonary disease; CPR, cardiopulmonary resuscitation; SD, standard deviation.

Appendix 7. Baseline characteristics post-ICU health problems

Baseline characteristics of patients with and without post-ICU health problems included in analysis (n = 2476).

Variable	Categories	Post-ICU health problems (N = 1568)	No post-ICU health problems (N = 681)	P value
Age, years – mean (SD)		61.40 (14.42)	61.07 (15.37)	.75
	Missing n/N (%)	87 (5.55)	22 (3.23)	
Gender – n/N (%)	Male	877 (55.93)	457 (67.11)	< .001
	Female	637 (40.63)	211 (30.98)	
	Missing	54 (3.44)	13 (1.91)	
Education level – n/N (%)	Low	538 (34.31)	171 (25.11)	< .001
	Middle	670 (42.73)	309 (45.37)	
	High	335 (21.36)	196 (28.78)	
	Missing	25 (1.59)	5 (0.73)	
Admission type – n/N (%)	Medical	754 (48.09)	339 (49.78)	.25
	Emergency surgery	245 (15.63)	90 (13.22)	
	Elective surgery	511 (32.59)	238 (34.95)	
	Missing	58 (3.70)	14 (2.06)	
Length of ICU stay – n/N (%)	<2 days	840 (53.57)	425 (62.41)	< .001
	≥ 2 days	674 (42.98)	243 (35.68)	
	Missing	54 (3.44)	13 (1.91)	
APACHE score– mean (SD)		55.62 (23.81)	55.53 (25.37)	.81
	Missing n/N (%)	87 (5.55)	22 (3.23)	
Comorbidities – n/N (%)	Chronic renal insufficiency	67 (4.27)	29 (4.26)	.93
	Chronic dialysis	11 (0.70)	11 (1.62)	.047
	Neoplasm	104 (6.63)	45 (6.61)	.91
	Immune insufficiency	212 (13.52)	60 (8.81)	< .001
	Hematologic malignancy	30 (1.91)	17 (2.50)	.40
	Cirrhosis	9 (0.57)	1 (0.15)	.30
	Cardiovascular insufficiency	36 (2.30)	10 (1.47)	.19
	Respiratory insufficiency	62 (3.95)	17 (2.50)	.074
	COPD	229 (14.60)	45 (6.61)	< .001
	Diabetes	185 (11.80)	67 (9.84)	.14
	Missing	54 (3.44)	13 (1.91)	
CPR – n/N (%)		55 (3.51)	55 (8.08)	< .001
	Missing	54 (3.44)	13 (1.91)	
Mechanical ventilation[ꝁ] – n/N (%)		794 (50.64)	346 (50.81)	.78
	Missing	54 (3.44)	13 (1.91)	
QoL (EQ-5D-5L) – median [IQR]		0.74 (0.48-0.88)	0.88 (0.70-1.0)	< .001
	Missing n/N (%)	87 (5.55)	22 (3.23)	

ꝁ Mechanical ventilation within first 24 hours of ICU admission

Abbreviation: APACHE, Acute Physiology and Chronic Health Evaluation; ICU, Intensive Care Unit; IQR, Interquartile range; COPD, chronic obstructive pulmonary disease; CPR, cardiopulmonary resuscitation; QoL, quality of life; SD, standard deviation.

Appendix 8. Formula

Formula of the PROSPECT model. Regression coefficients are based on internal validation.

$$P(y=1) = \frac{1}{1+\exp\left(-(\beta_0+\beta_1 x_1+\beta_2 x_2+\cdots)\right)}$$

$$P(y=1) = \frac{1}{1+\exp\left(-\left(\begin{array}{c} -0.860\ [intercept] \\ +\ 0.032\ score\ CIS\ [CIS] \\ +\ (0.438\ [urgent\ surgery\ admission] OR\ -0.335\ [planned\ \ surgery\ admission] OR\ 0\ [medical\ admission]) \\ +\ 0.392\ [LOS - ICU\ \geq\ 2days] \\ +\ 0.141\ [HADS - Anxiety\ score] \\ +\ 0.621\ [COPD] \end{array}\right)\right)}$$

Example:

A patient is admitted to the ICU with spinal cord decompression (urgent surgery admission). ICU physicians expect the ICU stay to last for at least 5 days. The patient has no COPD, and had a scores 28 on the CIS and 5 on the HADS-Anxiety. The probability (*P*) of developing post-ICU health problems 3 months after ICU admission can be calculated as follows:

$$P(y=1) = \frac{1}{1+\exp\left(-\begin{pmatrix} -0.860\ [intercept] \\ +\ 0.032 * 28\ [CIS] \\ +\ (0.438\ [urgent\ surgery\ admission]) \\ +\ 0.392 * 1\ [LOS - ICU\ \geq\ 2days] \\ +\ 0.141 * 5\ [HADS - Anxiety\ score] \\ +\ 0.621 * 0\ [COPD] \end{pmatrix}\right)} * 100\%$$

$$(P) = \frac{1}{1+\exp\left(-(-0.860+0.896+0.438+0.392+0.705+0)\right)} * 100\%$$

$$(P) = \frac{1}{1+\exp(-1.571)} * 100\%$$

$$(P) = 0.828 * 100\% = 83\%$$

Abbreviations: Length of ICU stay (LOS); Checklist individual Strength – fatigue subscale (CIS); Hospital Anxiety and Depression Scale (HADS); Chronic obstructive pulmonary disease (COPD).

Appendix 9. Bootstrap samples

Results of internal validation after 1000 bootstrap samples.

	Index.orig	Training	Test	Optimism	Index.corrected	n
**R2**	0.1857	0.1863	0.1857	0.0005	0.1852	1000
**Slope**	1.0000	1.0000	1.0044	-0.0044	1.0044	1000
**Intercept**	0.0000	0.0000	-0.0044	0.0044	-0.0044	1000
**B**	0.1821	0.1820	0.1824	-0.0005	0.1826	1000

Abbreviations: B, Brier; R2, R-squared; n, number.

₱ Positive score defined by a score of ≥5 on the Clinical Frailty Scale (CFS)

₫ Positive score defined by a score of >37 on the Checklist Individual Strength – fatigue subscale (CIS-8)

€ Positive score defined by a score of ≥ 8 on the Hospital Anxiety and Depression Scale (HADS) anxiety subscales

£ Positive score defined by a score of ≥ 8 on the Hospital Anxiety and Depression Scale (HADS) depression subscales

¥ Positive score defined as cognitive impairment with a score of ≥43 on the abbreviated Cognitive Failure Questionnaire (CFQ-14)

₭ Mechanical ventilation within first 24 hours of ICU admission

Appendix 10. Sensitivity and specificity plot of the PROSPECT model and cutoff points

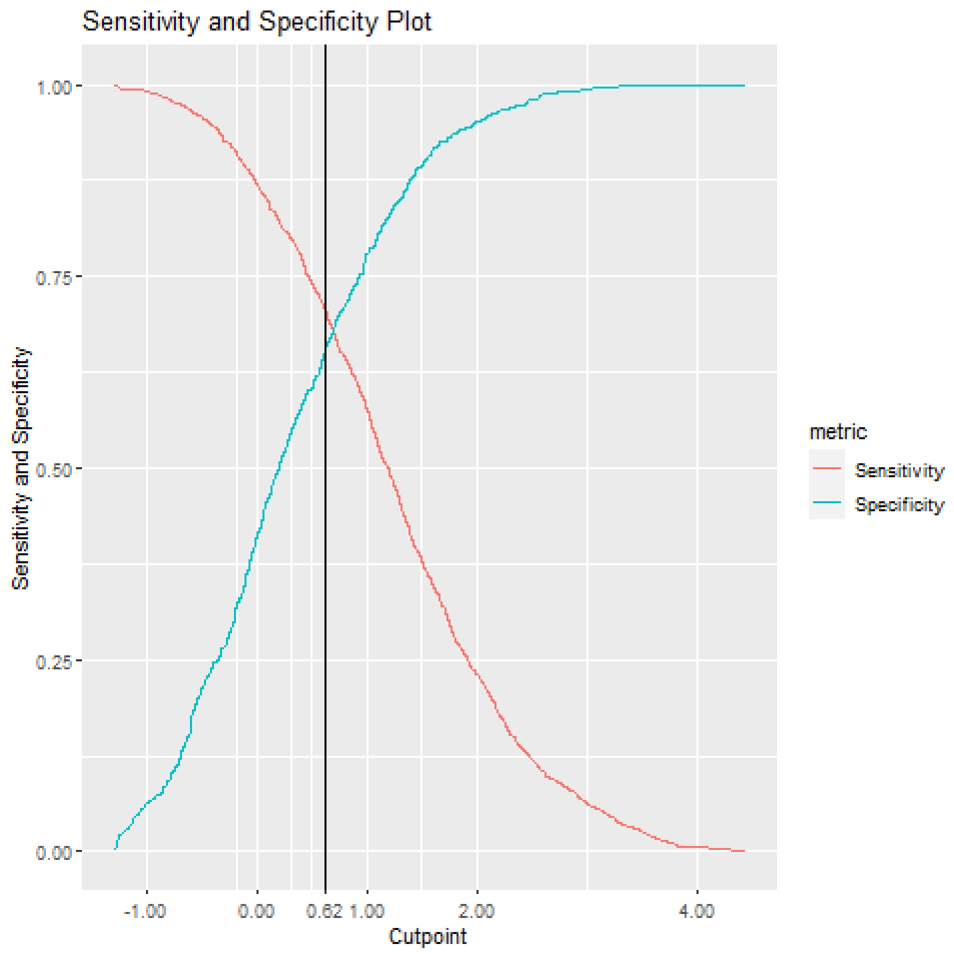

Appendix 11. Predicted probabilities

Density plot and histogram of the number of predicted probabilities (*100%) of the PROSPECT model for development and validation dataset.

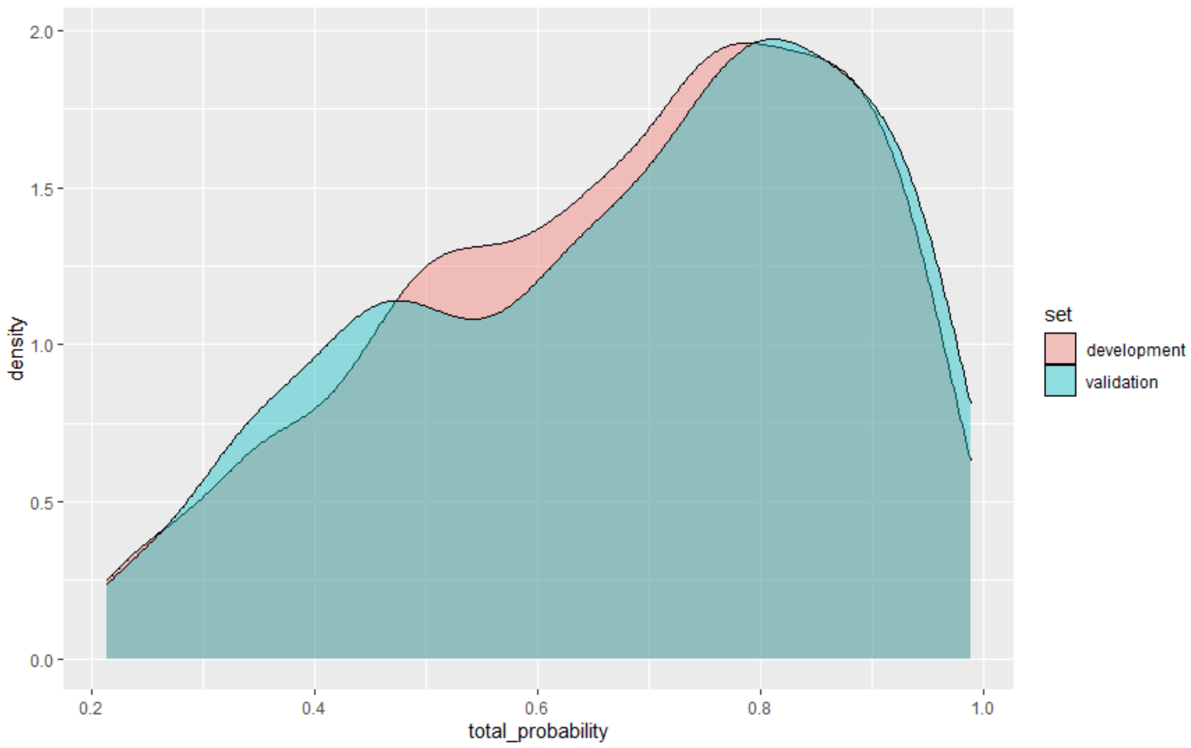

2.0
1.5
1.0
0.5
0.0
density
0.2
0.4
0.6
0.8
1.0
total_probability
set
development
validation

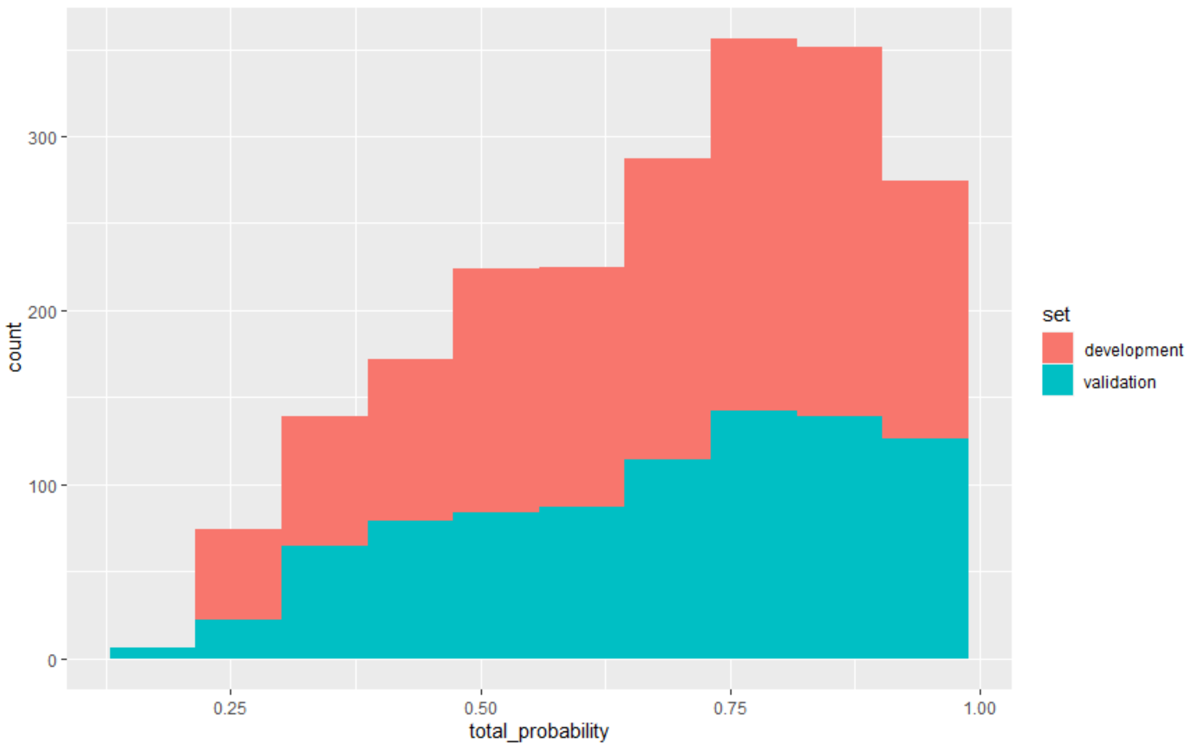
count
300
200
100
0
0.25
0.50
0.75
1.00
total_probability
set
development
validation

Appendix 12. Model development and performance without elective surgical patients.

**Variables**		**Regression coefficient**
Intercept		-0,25157
Admission type (ref. medical admission)	[emergency surgical]	0,378716
Frailty	[CFS categorised 1-9]	-0,12202
Anxiety	[score HADS-Anxiety]	0,217588
Fatigue	[score CIS]	0,020868
COPD	[yes/no]	0,687818
**Statistics**	**Internal validation**	**External validation**
Likelihood Chi-square	89.60	84.72
Nagelkerke R2	0.18	0.16
C-statistic (95% CI)	0.73 (0.69-0.77)	0.71 (0.67-0.75)
Brier score	0.17	0.20
Hosmer-Lemeshow X2 (P value)	20.09 (0.01)	120.03 (< .01)

Abbreviation: CI, Confidence Interval; CIS, Checklist individual Strength – fatigue subscale; CFS, Clinical Frailty Scale; COPD, chronic obstructive pulmonary disease; HADS, Hospital Anxiety and Depression scale; LR, likelihood ratio; Ref, reference category
